# Self-Reported Use of Personal Protective Equipment among Chinese Critical Care Clinicians during 2009 H1N1 Influenza Pandemic

**DOI:** 10.1371/journal.pone.0044723

**Published:** 2012-09-05

**Authors:** Xiaoyun Hu, Zhidan Zhang, Na Li, Dexin Liu, Li Zhang, Wei He, Wei Zhang, Yuexia Li, Cheng Zhu, Guijun Zhu, Lipeng Zhang, Fang Xu, Shouhong Wang, Xiangyuan Cao, Huiying Zhao, Qian Li, Xijing Zhang, Jiandong Lin, Shuangping Zhao, Chen Li, Bin Du, for the China Critical Care Clinical Trial Group (CCCCTG)

**Affiliations:** 1 Peking Union Medical College Hospital, Beijing, People's Republic of China; 2 The First Affiliated Hospital of China Medical University, Shenyang, People's Republic of China; 3 Hainan Provincial People's Hospital, Haikou, People's Republic of China; 4 The Second Hospital of Jilin University, Changchun, People's Republic of China; 5 Fuxing Hospital, Capital Medical University, Beijing, People's Republic of China; 6 Beijing Tongren Hospital, Capital Medical University, Beijing, People's Republic of China; 7 The First Affiliated Hospital of Kunming Medical College, Kunming, People's Republic of China; 8 The First Affiliated Hospital of Zhengzhou University, Zhengzhou, People's Republic of China; 9 Ruijin Hospital, Shanghai Jiaotong University, Shanghai, People's Republic of China; 10 Hebei Medical University Fourth Hospital, Shijiazhuang, People's Republic of China; 11 The Affiliated Hospital of Inner Mongolia Medical College, Huhhot, People's Republic of China; 12 The First Affiliated Hospital, Chongqing Medical University, Chongqing, People's Republic of China; 13 Guangdong General Hospital, Guangzhou, People's Republic of China; 14 Affiliated Hospital of Ningxia Medical University, Yinchuan, People's Republic of China; 15 Peking University People's Hospital, Beijing, People's Republic of China; 16 Zhejiang Provincial People's Hospital, Hangzhou, People's Republic of China; 17 Xijing Hospital, Xi'an, People's Republic of China; 18 The First Affiliated Hospital of Fujian Medical University, Fuzhou, People's Republic of China; 19 Xiangya Hospital, Central South University, Changsha, People's Republic of China; 20 Qilu Hospital of Shandong University, Jinan, People's Republic of China; Barcelona University Hospital, Spain

## Abstract

**Background:**

Critically ill patients with 2009 H1N1 influenza are often treated in intensive care units (ICUs), representing significant risk of nosocomial transmission to critical care clinicians and other patients. Despite a large body of literature and guidelines recommending infection control practices, numerous barriers have been identified in ICUs, leading to poor compliance to the use of personal protective equipment (PPE). The use of PPE among critical care clinicians has not been extensively evaluated, especially during the pandemic influenza. This study examined the knowledge, attitudes, and self-reported behaviors, and barriers to compliance with the use of PPE among ICU healthcare workers (HCWs) during the pandemic influenza.

**Methodology/Principal Findings:**

A survey instrument consisting of 36 questions was developed and mailed to all HCWs in 21 ICUs in 17 provinces in China. A total of 733 physicians, nurses, and other professionals were surveyed, and 650 (88.7%) were included in the analysis. Fifty-six percent of respondents reported having received training program of pandemic influenza before they cared for H1N1 patients, while 77% reported to have adequate knowledge of self and patient protection. Only 18% of respondents were able to correctly identify all components of PPE, and 55% reported high compliance (>80%) with PPE use during patient care. In multivariate analysis, vaccination for 2009 H1N1 influenza, positive attitudes towards PPE use, organizational factors such as availability of PPE in ICU, and patient information of influenza precautions, as well as reprimand for noncompliance by the supervisors were associated with high compliance, whereas negative attitudes towards PPE use and violation of PPE use were independent predictors of low compliance.

**Conclusion/Significance:**

Knowledge and self-reported compliance to recommended PPE use among Chinese critical care clinicians is suboptimal. The perceived barriers should be addressed in order to close the significant gap between perception and knowledge or behavior.

## Introduction

On April 29, 2009, the World Health Organization (WHO) announced the outbreak of a novel influenza A (H1N1) 2009 virus to be a public health emergency of international concern [1], which ultimately led to the declaration of the first phase 6 global influenza pandemic on June 11, 2009 [2]. As of September 6, 2009, the WHO had reported more than 277,607 laboratory-confirmed cases, with at least 3,205 deaths [3]. Studies estimated that up to 2.7 million patients would be hospitalized, and about 25% of these patients might experience rapid deterioration, leading to intensive care unit (ICU) admission within 1 day after hospitalization, equivalent to an increase in the volume of mechanical ventilation of 23% to 45% over the current use [4], [5]. All these data suggested an excessive workload during the initial period of the pandemic, as perceived by 80% of frontline healthcare workers (HCWs) [6].

A simulation study by Swaminathan and the colleagues reported that, for a patient with suspected avian or pandemic influenza who was not clinically unwell or hypoxic, the mean number of close contacts was 12.3 (range 6–17; 85% HCWs), and mean exposures were 19.3 (range 15–26) during the first 6 hours in the emergency departments [7]. In comparison, critical care clinicians are likely to encounter even more repeated close contacts, and are at significantly high risk of acquiring such an infectious disease during patient care. Evidence does exist suggesting nosocomial transmission within hospital settings. Apart from earlier findings that more than 20% of patients who acquired severe acute respiratory syndrome (SARS) were HCWs [8], possible healthcare-related 2009 H1N1 influenza transmission was identified in 9 out of 63 exposed HCWs [9].

Protection of HCWs from acquisition of infectious diseases can be achieved by compliance to established infection control guidelines [10]–[13], including rigorous infection control practices, prescriptive instructions for the use of personal protective equipment (PPE), and postexposure antiviral prophylaxis [7]. However, reported compliance to PPE use might be extremely low. In response to a survey conducted by the Center for Disease Control and Prevention following the pandemic influenza, among 11 HCWs with probable or possible patient-to-HCW transmission, only 3 reported always using either a surgical mask or an N95 respirator [14]. A variety of barriers have been identified to hinder compliance to infection prevention and control guidelines, including knowledge, attitude, belief and behavioral factors [15].

Daugherty and colleagues explored the behavior, knowledge, and attitudes of critical care clinicians about recommended precautions for prevention of healthcare-associated influenza infections in an anticipated influenza pandemic [16]. With the same methodology using a modified questionnaire, we previously reported that 82.3% of the ICU HCWs expressed willingness to work in a pandemic, with professions, knowledge training prior to patient care, and the confidence to know how to protect themselves and the patients independently associated with more likelihood to care for H1N1 patients [17]. However, little is known about their behavior and factors influencing compliance during a real influenza pandemic. As the second part of the above survey, we wish to evaluate the self-reported compliance to the use of PPE during the current influenza pandemic among critical care clinicians in Chinese ICUs, as well as independent predictors of the compliance.

## Methods

This study was approved by the institutional review board (IRB) of Peking Union Medical College Hospital. All participants were informed about the study. However, the IRB waived the need for written informed consent from the participants because the identities of all respondents would be completely anonymous during data collection and analysis, and there would be minimal risk as perceived by the IRB for being involved in this study.

The design of this study was described in details elsewhere [17]. In brief, this study was conducted in 21 adult ICUs in 17 provinces in China. All participating ICUs admitted patients with 2009 H1N1 influenza during the pandemic. A 36-item survey questionnaire was designed based on the study of Daugherty and coworkers [16], to assess the knowledge, attitudes, and behaviors of ICU HCWs related to the 2009 H1N1 influenza pandemic, which was available as supporting information; see **Questionnaire S1.** On December 25, 2009, the questionnaire with an instruction was sent by e-mail to the contact persons of individual participating ICUs, who encouraged as many HCWs as possible to participate the study. All questionnaires were collected and sent back by e-mail before January 15, 2010. Any HCWs not responding after the deadline were regarded as non-respondents.

Data on the demographic characteristics of respondents, including age, sex, marital status, living status, status of influenza vaccination, and profession, were recorded. The professional status of the respondents was categorized as physicians, and nurses, and others (including respiratory therapists, student nurses, and nurse assistants). For the purpose of this study, we only included physicians and nurses in the final analysis.

The respondents were asked to report their experience of caring for H1N1 patients, as well as relevant training. They were also required to report the level of knowledge and the level of confidence in their ability to protect themselves and their patients from exposure to influenza at work. A 5-point Likert scale (complete agree, agree, neither agree nor disagree, disagree, and complete disagree) was used to elicit preferred answers. We defined recommended PPE as use of hand hygiene, gloves, gown, mask (including surgical mask and N95 respirator), and goggles [13]. In the final analysis, answers with a higher level of protection than recommended (e.g. use of goggles when no aerosol-generating procedures were anticipated) were deemed as correct because they represented adequate protection [16].

As a response to the 2009 H1N1 influenza pandemic, all hospitals were required by local healthcare authorities to provide training programs to all hospital staffs during seminars. These training programs were mainly 2 to 3-hour lectures, developed based on the guidelines issued by Ministry of Health, often involving diagnosis, treatment, and infection control of 2009 H1N1 influenza. There was no posttest to evaluate the extent of information attainment by the attendees.

### Statistical Analysis

All Likert-scale responses were dichotomized into complete agree/agree versus neither agree nor disagree/disagree/disagree/complete disagree, and expression in proportions. Continuous variables were compared with Student's t-test or Mann-Whitney U test. Categorical variables were compared with chi-square test or Fisher's exact test when appropriate. Self-reported compliance to PPE use of >80% was considered as high compliance [16]. Correlations were measured using Kendall rank correlation coefficient. For determination of independent predictors for high compliance to PPE use during patient care, odds ratio (OR) was estimated on the basis of both univariate analysis and multivariate logistic regression analysis. Variables including clinicians characteristics, knowledge, attitudes, and behaviors were added into the model using stepwise conditional forward entry, if p<0.1 in univariate analysis. An OR of less than 1 was associated with low compliance to PPE use, while an OR of greater than 1 was associated with high compliance to PPE use during patient care.

## Results

### Respondents

In the 21 ICUs surveyed, 733 eligible participants were identified, and 695 returned completed surveys, for an overall response rate of 94.8%. Forty-five respondents were excluded (including 25 other professionals, and 20 with missing data), therefore 650 respondents (including 229 physicians and 421 nurses) were included in the final analysis (Table 1). Compared with physicians, more nurses were single, and living with parents or living alone. More than half respondents received vaccination for 2009 H1N1 influenza. Five hundred and eighty-six respondents (90.2%) reported that they had received the pandemic training program, although only 364 (56.0%) claimed to complete the pandemic training program before they cared for H1N1 patients.

**Table 1:** Characteristics of respondents.

	Physicians (n = 229)	Nurses (n = 421)	Total (n = 650)
Age, median [interquartile range]***	32 [28 to 38]	27 [24 to 30]	28 [25 to 33]
Male sex***	56.8% (130)	5.7% (24)	23.7% (154)
Married***	76.0% (174)	44.2% (186)	55.4% (360)
Living status***			
With parents	24.0% (55)	31.1% (131)	28.6% (186)
With children	35.8% (82)	12.8% (54)	20.9% (136)
With spouse only	20.1% (46)	21.4% (90)	20.9% (136)
Alone	20.1% (46)	34.7% (146)	26.5% (172)
Vaccination for seasonal influenza	4.4% (10)	5.5% (23)	5.1% (33)
Vaccination for 2009 H1N1 influenza*	47.6% (109)	56.1% (236)	53.1% (345)
I have finished the pandemic training program*	93.9% (215)	88.1% (371)	90.2% (586)
I have finished the pandemic training program before I cared for H1N1 patients	56.8% (130)	55.6% (234)	56.0% (364)

* p<0.05, **p<0.01, ***p<0.001, physicians versus nurses.

### Knowledge

Significantly more physicians than nurses reported to have adequate knowledge of 2009 H1N1 influenza (61.1% vs. 33.7%, p<0.001). The most commonly identified component of PPE was hand hygiene (95.4%), followed by gloves (90.0%), gown (88.6%), N95 respirator (88.3%), and goggles (81.4%). However, only 215 respondents (33.1%) believed that surgical mask provided adequate protection under certain circumstances. These resulted in all components of PPE correctly identified by 116 respondents (17.8%). Moreover, among 435 respondents who considered surgical mask as inadequate PPE for pandemic influenza, 426 (97.9%) identified N95 respirator as appropriate, indicating the source of overprotection. A similar proportion of respondents (18.8%) exhibited adequate knowledge of hand hygiene. The item with the least correct answer (22.2%) was that alcohol handrub could not be used when hands were visibly soiled or contaminated. In comparison, about three-fourths of respondents reported to wear goggles and gown during aerosol-generating procedures, and to wear N95 respirator in droplet precaution or close contact, respectively. However, 435 respondents (66.9%) reported to wear goggles and gown during entire treatment and/or nursing care, indicating overprotection. Significant correlation was found between self-reported adequate knowledge of pandemic influenza and correct identification of PPE and knowledge of goggles (Kendall tau-b 0.184 and 0.131, p<0.001 and p = 0.001, respectively), but not knowledge of hand hygiene or mask (Kendall tau-b −0.007 and −0.029, p = 0.851 and 0.467, respectively).

### Attitude

About 80% of respondents believed that they knew self- and patient protection during the pandemic (Table 2). In particular, 87.5% of respondents believed that use of appropriate PPE would confer adequate protection for HCWs, while only 68.5% stated that this protection was adequate for vulnerable patients. Half of respondents reported that PPE use was inconvenient, while 21.2% believed that PPE use would interfere with patient care, with no difference observed between physicians and nurses. No significant correlation was found between self-reported adequate knowledge of both self-protection and patient protection and correct knowledge of hand hygiene, goggles, or masks. However, self-reported adequate knowledge was significantly correlated with the perception of further improvement of PPE compliance (Kendall tau-b 0.143, p<0.001).

**Table 2:** Use of PPE during 2009 influenza pandemic: knowledge, attitudes, and behaviors.

	Physicians (n = 229)	Nurses (n = 421)	Total (n = 650)
Knowledge			
I have the knowledge of H1N1 influenza***	61.1% (140)	33.7% (142)	43.4% (282)
Correct identification of PPE	19.7% (45)	16.9% (71)	17.8% (116)
Correct knowledge of hand hygiene	20.5% (47)	17.8% (75)	18.8% (122)
When to wear goggles and gown during patient care	71.6% (164)	75.8% (319)	74.3% (483)
When to wear mask during patient care**	65.9% (151)	77.0% (324)	73.1% (475)
Attitudes			
I am confident that I understand the risks of a pandemic for patients and HCWs	83.8% (192)	79.3% (334)	80.9 (526)
I am confident that I know how to protect myself and my patients during a pandemic	81.2% (186)	74.6% (314)	76.9% (500)
Use of PPE will keep HCWs from getting H1N1 influenza	85.6% (196)	88.6% (373)	87.5% (569)
Use of PPE will keep patients from getting H1N1 influenza**	61.6% (141)	72.2% (304)	68.5% (445)
PPE use inconvenient	47.1% (108)	51.3% (216)	49.8% (324)
PPE use interfere with patient care	20.1% (46)	21.9% (92)	21.2% (138)
I am confident that I can improve PPE compliance	75.1% (172)	74.1% (312)	74.5% (484)
Behaviors and management			
PPE readily available in ICU	59.4% (136)	65.3% (275)	63.2% (411)
I would be reprimanded by a supervisor*	86.4% (198)	92.1% (388)	90.2% (586)
Knowledge of patients on influenza precautions*	87.8% (201)	80.3% (338)	82.9% (539)
My colleagues often forget to use PPE during patient care	22.3% (51)	20.4% (86)	21.1% (137)
I would remove PPE immediately after leaving patient room*	81.2% (186)	72.4% (305)	75.5% (491)
I often forget to change PPE between patients	22.3% (51)	21.4% (90)	21.7% (141)
High compliance	50.7% (116)	58.2% (245)	55.5% (361)

HCW, healthcare worker; ICU, intensive care unit; PPE, personal protective equipment.

* p<0.05, **p<0.01, ***p<0.001, physicians versus nurses.

### Behaviors

With regards to organization factors, 63.2% of respondents reported that appropriate PPE was readily available in their ICUs (Table 2). More physicians than nurses knew when influenza precautions were initiated in their patients (p = 0.015). By contrast, significantly more nurses than physicians (92.1% vs. 86.4%, p = 0.020) reported being reprimanded by the supervisor for noncompliance. As to behaviors of PPE use, about 21% of respondents reported that their colleagues often forgot to use PPE during patient care, while a similar proportion reported themselves to forget to change PPE between patients.

### Predictors

Among all respondents, 361 (55.5%) reported high compliance (>80%) to PPE use, with significant inter-institutional variation ranging from 0% (0/5) to 88.1% (37/42). The independent factors of high compliance to PPE use included vaccination for 2009 H1N1 influenza (OR 1.940, 95% confidence interval [CI] 1.357–2.774, p<0.001), positive attitudes towards PPE use (belief that use of recommended PPE would confer adequate protection for HCWs) (OR 2.696, 95%CI 1.520–4.782, p = 0.001), availability of PPE in ICU (OR 1.609, 95%CI 1.086–2.385, p = 0.018), recognition of patients on influenza precaution (OR 2.051, 95%CI 1.260–3.336, p = 0.004), and perceived reprimand by the supervisor for noncompliance (OR 1.972, 95%CI 1.048–3.709, p = 0.035). Negative attitudes towards PPE use (perception that PPE use would interfere with patient care) (OR 0.455, 95%CI 0.274–0.755, p = 0.002), and violation of recommended infection control measures (forgetting to use PPE [OR 0.361, 95%CI 0.225–0.581, p<0.001] or change PPE [OR 0.342, 95%CI 0.213–0.550, p<0.001]) were independently associated with self-reported low compliance (Table 3).

**Table 3:** Predictors of high compliance to PPE use among ICU clinicians: results of univariate and multivariate analyses.

	Univariate analysis OR (95%CI)	Multivariate analysis OR (95%CI)
Profession		
Physician	Ref.	
Nurse	1.356 (0.981–1.874)	
Living status		
Alone	Ref.	
With parents	1.557 (1.033–2.346)	
With children	1.302 (0.836–2.027)	
With spouse only	0.912 (0.588–1.416)	
Vaccination for 2009 H1N1 influenza	1.756 (1.284–2.400)	1.940 (1.357–2.774)
I have finished H1N1 training program before I cared for H1N1 patients	1.384 (1.013–1.890)	
I have the knowledge of H1N1 influenza	1.371 (1.002–1.877)	
Correct identification of PPE	1.738 (1.141–2.648)	
Correct knowledge of hand hygiene	0.701 (0.472–1.040)	
When to wear goggles and gown during patient care	1.372 (0.964–1.953)	
When to wear mask during patient care	1.469 (1.037–2.080)	
Attitudes		
Use of PPE will keep HCWs from getting H1N1 influenza	3.690 (2.213–6.152)	2.696 (1.520–4.782)
Use of PPE will keep patients from getting H1N1 influenza	2.238 (1.598–3.135)	
Use of PPE inconvenient	0.742 (0.544–1.012)	
Use of PPE interfere with patient care	0.519 (0.355–0.760)	0.455 (0.274–0.755)
Willingness to care H1N1 influenza patients	3.483 (2.234–5.431)	
Behaviors and management		
PPE readily available in ICU	2.781 (2.002–3.863)	1.609 (1.086–2.385)
I would be reprimanded by a supervisor	2.620 (1.525–4.502)	1.972 (1.048–3.709)
Knowledge of patients on influenza precautions	2.614 (1.711–3.994)	2.051 (1.260–3.336)
My colleagues often forget to use PPE during patient care	0.247 (0.164–0.371)	0.361 (0.225–0.581)
I often forget to change PPE between patients	0.222 (0.147–0.335)	0.342 (0.213–0.550)
I am confident that I can improve PPE compliance	1.440 (1.011–2.052)	

## Discussion

To our knowledge, this study represents the first effort to examine self-reported knowledge, attitude, behavior and influencing factors of PPE use during the pandemic influenza in Chinese ICUs. Among 650 respondents, although up to 77% reported to have adequate knowledge of self- and patient protection, fewer than 20% could correctly identify all components of PPE or exhibited correct knowledge of PPE use during patient care. This suggested significant gaps in the perception and actual knowledge with regards to infection control practices, in particular PPE use, among our critical care clinicians [17]. Moreover, about 55% of respondents reported high compliance to the recommended PPE use. Vaccination status, positive attitudes towards PPE use, cultural factor (perceived reprimand for noncompliance), and organizational factors (availability of PPE in ICU, notice of influenza precautions) were identified as independent predictors of high compliance, while negative attitudes towards PPE use and violation of recommended PPE use were associated with low compliance.

PPE referred to a variety of barriers and respirators used alone or in combination to protect mucous membranes, airways, skin, and clothing from contact with infectious agents [12]. The critical importance of compliance to PPE use was not only recognized in a variety of practice guidelines of infection control [9]–[13], but also demonstrated during the outbreak of SARS in 2003 [8], [18]. Unfortunately, compliance by professionals was often suboptimal [19], [20], due to knowledge, attitudes, and behavior among professionals, as well as to organizational and other factors [15], [21]. In this survey of Chinese critical care clinicians, only 55% of respondents reported high compliance (>80%) to recommended PPE use, consistent with other relevant studies [16], [19]. However, significant gaps between perception and practice were a common finding in ICU [22], indicating overestimation of clinical practice judged by self-reported behavior, especially for infection control measures, such as hand hygiene [23] and PPE use [16], [19]. Similar to the study of Daugherty and coworkers [16], we found a similar proportion (74%) of respondents claiming their confidence to improve compliance to PPE use, again suggesting perception of inadequate PPE use among most respondents.

Our results indicated that a number of factors, including attitudes, behavior, and organization, might significantly influence clinical practice. Although behavior could be changed without knowledge or attitude being affected, behavior change (i.e. self-reported high compliance to PPE use) based on improving knowledge and attitude (e.g. PPE use could confer adequate protection for HCWs) was probably more sustainable than indirect manipulation of behavior alone [15]. In the meanwhile, it was also self-intuitive that a negative attitude (e.g. perception that PPE use might interfere with patient care) often predicted low compliance. Likewise, Daugherty and coworkers found that the belief that PPE use was inconvenient was predictive of poorer adherence [16]. The perception that PPE use interfered with patient care was supported by previous studies. Despite the fact that critical care clinicians were probably highly compliant with PPE use, patients in contact isolation might suffer from adverse effect of inadequate patient care, including less time spent in patient rooms not explained by severity of illness [24], [25], less time examining patients [26], more incomplete records of vital signs and progress notes, and increasingly likelihood of preventable adverse events. Moreover, almost half HCWs reported difficulty in communicating with patients through enhanced infection precautions during the SARS outbreak [27].

Organizational factors were commonly acknowledged as barriers that impede and hamper professionals' compliance to PPE. Compliance to PPE use was closely related to the professionals' perception about the risks they were exposed to and their susceptibility to these risks. Our study showed that, if critical care clinicians were aware of the patients on isolation precautions, they were twice likely to report high compliance to PPE use. Similarly, in a survey of physicians working in Canadian pediatric emergency departments, almost 90% considered identifying patients with complaints requiring PPE use prior to the physician entering the room as an important factor promoting PPE use [19]. In a study performed during the first wave of 2009 H1N1 influenza, Banach and coworkers observed more unprotected exposures in patients who did not present with influenza-like illness [28]. This finding was not unexpected because such patients would not have been identified by the screening protocol, which might result in delays in consideration of influenza as a potential diagnosis when these patients were subsequently evaluated by clinicians, as well as delays in implementation of recommended infection control measures.

Studies have consistently demonstrated significant association of the availability of PPE in ICU and self-reported compliance, as in our study, indicating unavailability as the major reason for non-compliance [16], [19], [21]. However, among the 256 critical care clinicians surveyed by Daugherty and coworkers, self-reported high compliance was only 62%, despite the fact that 72% reported that recommended PPE was readily available near patients' rooms [16]. This evidenced the complexity of compliance to PPE, which might go beyond availability, confirming the interference of individual factors, perceptions, and relations in the work environment in decision making towards protection.

Professional's behavior was an important factor that determined the commitment to, and the style and proficiency of, an organization's health and safety management [29]. A study in 1986 examined the role of organizational factors in 13 hospitals in the United States, and found that severity-adjusted mortality were related more to the interaction and coordination of each hospital's ICU staff than the ICU administrative structure, amount of specialized treatment used, or the hospital's teaching status [30]. Similar to other studies [16], our study found close association between self-reported compliance and safety culture (i.e. HCW behavior, and perceived reprimand for noncompliance by the supervisors), underscoring the importance of ICU safety culture in promoting behavior change, or even patient outcome [29].

Perceived barriers of compliance to PPE use as described above should be addressed during development of practice guidelines, in order to prevent transmission of infectious diseases within hospital setting. Despite the lack of data validating such concept with regards to 2009 H1N1 influenza in ICU, studies did suggest that implementation of protocoled care and/or educational program, by addressing knowledge, attitude, and behavioral barriers, might significantly reduce catheter-related bloodstream infection [31], and improve mortality in patients with severe sepsis [32].

The major limitation of our study was that it might be subject to social desirability bias (individuals may wish to present themselves or their organization in a favorable way) due to its reliance on self-reporting [33]. In addition, cause-effect relationship could not be determined due to the inherent “chicken or egg” caveat of the observational study. Nevertheless, these data provided clue of the barriers that existed with regard to the implementation of infection control guidelines in ICUs and provided useful suggestions for the implementation.

## Conclusions

Only 55% of Chinese critical care clinicians reported high compliance to PPE use during pandemic influenza, putting HCWs and their patients at risk. Both attitudes towards PPE use and perceived organizational norms have been recognized as predictors of compliance, which should be addressed while developing educational program and/or practice guidelines, in order to prevent nosocomial transmission of influenza.

## Supporting Information

Questionnaire S1 Survey Questionnaire.
(DOC)
