# Supplementary material for: Self-Reported Use of Personal Protective Equipment among Chinese Critical Care Clinicians during 2009 H1N1 Influenza Pandemic
Source: PLoS One. 2012 Sep 5;7(9):e44723. doi: 10.1371/journal.pone.0044723 (PMC3434157; doi:10.1371/journal.pone.0044723)
Supplement: Questionnaire S1 — Survey Questionnaire. (DOC) [file pone.0044723.s001.doc]

**Additional File**

**Self-Reported Use of Personal Protective Equipment Among Chinese Critical Care Clinicians During 2009 H1N1 Influenza Pandemic**

**Questionnaire S1 Survey Questionnaire**

Demographics

1. Name of Hospital
2. Age
3. Sex
   1. Male
   2. Female
4. Marital Status
   1. Single
   2. Married
   3. Other (please specify: _________)
5. Professional
   1. Physician
   2. Nurse
   3. Other (please specify: _________)
6. Living Status
   1. Living with parents
   2. Living with children
   3. Living with spouse only
   4. Living alone

Status of Vaccination

1. Have you received vaccination for seasonal influenza in 2008-2009?
   1. Yes
   2. No
2. Have you received vaccination for 2009 A/H1N1 influenza in 2009?
   1. Yes
   2. No
3. If you did not receive vaccination for 2009 A/H1N1 influenza, what is the major reason? (multiple choices are allowed)
   1. Majority of patients with H1N1 influenza experience mild and self-limited course of disease
   2. I have contraindication for vaccination
   3. I have serious concern about the safety of H1N1 vaccination
   4. I have serious concern about the efficacy of H1N1 vaccination
   5. H1N1 vaccination is not available
   6. Other (please specify: ________)

Work with H1N1 patients

1. Have you had the experience treating or caring for patients with H1N1 influenza?
   1. Yes
   2. No
2. Have you finished the training program about H1N1 influenza?
   1. Yes
   2. No
3. Have you finished the training program about H1N1 influenza before caring for patients with H1N1?
   1. Yes
   2. No

Knowledge

1. You understand the relevant knowledge of H1N1 influenza.
   1. Complete agree
   2. Agree
   3. Neither agree nor disagree
   4. Disagree
   5. Complete disagree
2. The source of your knowledge about H1N1 influenza include: (multiple choices are allowed)
   1. Television
   2. Newspaper
   3. Internet
   4. Medical journals
   5. Hospital training program
   6. Other (please specify: ________)
3. You are confident that you understand the risks of H1N1 influenza pandemic for the patients and healthcare workers.
   1. Complete agree
   2. Agree
   3. Neither agree nor disagree
   4. Disagree
   5. Complete disagree
4. You are confident that you understand how to protect yourself and your patients during H1N1 influenza pandemic.
   1. Complete agree
   2. Agree
   3. Neither agree nor disagree
   4. Disagree
   5. Complete disagree
5. Correct personal protective equipments (PPE) include (multiple choices are allowed)
   1. Surgical mask
   2. N95 mask
   3. Gown
   4. Gloves
   5. Goggles
   6. Hand hygiene
   7. None of above
6. Hand hygiene includes either washing hands with soap and water, or the use of an alcohol-based hand rub.
   1. Complete agree
   2. Agree
   3. Neither agree nor disagree
   4. Disagree
   5. Complete disagree
7. Wash hands with an alcohol-based hand rub when they are visibly soiled.
   1. Complete agree
   2. Agree
   3. Neither agree nor disagree
   4. Disagree
   5. Complete disagree
8. Use of correct PPE eliminates the need for hand hygiene.
   1. Complete agree
   2. Agree
   3. Neither agree nor disagree
   4. Disagree
   5. Complete disagree
9. When should you wear an eye protection (i.e. goggles or a face shield) and a clean, non-sterile, long-sleeved gown during care for patients with H1N1 influenza? (multiple choices are allowed)
   1. During entire treatment and/or nursing care
   2. When performing aerosol-generating procedures associated with an increased risk of infection transmission
   3. During activities that are likely to generate splashes or sprays of blood, body fluids, secretions, and excretions
   4. When performing aspirating or open suctioning of the lower respiratory tract
   5. When performing endotracheal intubation
   6. When performing CPR
   7. When performing bronchoscopy
   8. Other (please specify: ________)
10. When should you wear a surgical mask or N95 mask during care for patients with H1N1 influenza? (multiple choices are allowed)
    1. During entire treatment and/or nursing care
    2. If you are working within approximately 1 meter of a patient with H1N1 influenza
    3. When the patient is on droplet precautions
    4. Other (please specify: ________)

Attitudes

1. Use of PPE will keep healthcare workers from getting H1N1 influenza
   1. Complete agree
   2. Agree
   3. Neither agree nor disagree
   4. Disagree
   5. Complete disagree
2. Use of PPE will keep patients from getting H1N1 influenza
   1. Complete agree
   2. Agree
   3. Neither agree nor disagree
   4. Disagree
   5. Complete disagree
3. It is inconvenient to use recommended PPE when taking care for patients with H1N1 influenza
   1. Complete agree
   2. Agree
   3. Neither agree nor disagree
   4. Disagree
   5. Complete disagree
4. Use of recommended PPE interfere with patient treatment and/or nursing care
   1. Complete agree
   2. Agree
   3. Neither agree nor disagree
   4. Disagree
   5. Complete disagree
5. Are you willing to treat and/or care for patients with H1N1 influenza if you have the opportunity?
   1. Yes
   2. No
6. If you choose “no” to the above question, what is the major reason? (multiple choices are allowed)
   1. Concern about the possible infection of yourself
   2. Concern about the possible infection of your family members
   3. Other (please specify: ________)

Behaviors and Management

1. All recommended PPE is readily available in the ICU
   1. Complete agree
   2. Agree
   3. Neither agree nor disagree
   4. Disagree
   5. Complete disagree
2. Your head nurse or attending physician would reprimand you if you did not use PPE when caring for patients with H1N1 influenza
   1. Complete agree
   2. Agree
   3. Neither agree nor disagree
   4. Disagree
   5. Complete disagree
3. You know when your patients are on influenza precautions
   1. Complete agree
   2. Agree
   3. Neither agree nor disagree
   4. Disagree
   5. Complete disagree
4. Your colleagues often forget to use recommended PPE when taking care of patients with H1N1 influenza
   1. Complete agree
   2. Agree
   3. Neither agree nor disagree
   4. Disagree
   5. Complete disagree
5. The estimated compliance to recommended PPE during treatment and/or care of patients with H1N1 influenza is:
   1. 0%
   2. 10%
   3. 20%
   4. 30%
   5. 40%
   6. 50%
   7. 60%
   8. 70%
   9. 80%
   10. 90%
   11. 100%
6. You will remove your PPE immediately when you leave the patients room
   1. Complete agree
   2. Agree
   3. Neither agree nor disagree
   4. Disagree
   5. Complete disagree
7. You often forget to change PPE between patients when taking care of patients with H1N1 patients
   1. Complete agree
   2. Agree
   3. Neither agree nor disagree
   4. Disagree
   5. Complete disagree
8. You believe that you can improve the compliance to recommended PPE
   1. Complete agree
   2. Agree
   3. Neither agree nor disagree
   4. Disagree
   5. Complete disagree
